# Fatigue und sensomotorische Instabilität: Neurologisch kontrollierte Konversion von Post-COVID-19-Patienten

**DOI:** 10.1007/s00115-024-01732-3

**Published:** 2024-09-18

**Authors:** Thomas Urban, Fritjof Reinhardt, Peter Lohse, Stefan G. Spitzer, Luise Rasche, Heinz Reichmann

**Affiliations:** 1 Hochschule Schmalkalden, Schmalkalden, Deutschland; 2 BTU Cottbus-Senftenberg, Brandenburgische Technische Universität Cottbus Senftenberg, Cottbus-Senftenberg, Deutschland; 3 Medizinische Fakultät Carl Gustav Carus, Technische Universität Dresden, Dresden, Deutschland; 4 Klinik für Innere Medizin und Kardiologie, Herzzentrum Dresden, Dresden, Deutschland; 5 Dr.-D.-Erxleben-Str. 2a, 01968 Senftenberg, Deutschland

**Keywords:** Post-COVID-Syndrom, Fatigue, Sensomotorische Instabilität, Kognitive Verhaltenstherapie, Sekundäre psychosomatische Symptome, Post-COVID syndrome, Fatigue, Sensorimotor instability, Cognitive behavioral therapy, Secondary psychosomatic symptoms

## Abstract

**Hintergrund:**

Für die Behandlung der Symptome des Post-COVID-19-Syndroms wird derzeit keine kausale Therapie nach evidenzbasierten Kriterien breit empfohlen. Die Evaluation der Veränderungen des Leitsymptoms Fatigue und der sensomotorischen Instabilität durch eine individualisierte beanspruchungsgesteuerte Trainingstherapie sowie durch eine intensivierte kognitive Verhaltenstherapie war das übergeordnete Ziel der Interventionsstudie über einen 3‑jährigen Zeitraum (Q1-2021 bis Q4-2023).

**Methodik:**

Es wurden in den 3 Jahren im Post-COVID-19-Zentrum Lausitz (Senftenberg) 407 geimpfte nukleokapsidpositive Patienten behandelt. Bei 78 (rd. 19 %) von ihnen wurden als Leitsyndrome Fatigue/immunometabolische Depression und sensomotorische Instabilität konstatiert. Die Evaluation der individualisierten beanspruchungsgesteuerten Trainingstherapie erfolgte anhand der konkreten Post-COVID-19-Syndromatik und motorischer Fatigability-Parameter. Die sekundäre psychosomatische Syndromatik wurde mit kognitiven Fatigability-Parametern und mit Instrumenten der kognitiven Verhaltenstherapie bewertet. Die Untersuchung verhaltensbeeinflussender Parameter fand in Q2-2023 bis Q4-2023 mit einem leitfadengestützten qualitativen Interview unter den Therapieteilnehmern statt.

**Ergebnisse:**

Die Post-COVID-19-Leitsymptomen „Fatigue“, „sensomotorische Instabilität“, „neuropsychiatrische Symptome“, „kardionale/autonome Dysfunktion“ und „Schmerzen“ verbesserten sich signifikant in der Gesamtkohorte sowie bei der geschlechtsspezifischen Analyse. Eine Verschlechterung trat bei „sekundären psychosomatischen Symptomen“ auf. Für alle motorischen Fatigability-Parameter konnte für die Gesamtkohorte mit dem Cohens d-Wert ein therapeutischer Effekt nachgewiesen werden. Positive Wirkungen erzielte eine Intensivierung der kognitiven Verhaltenstherapie durch eine zunehmende Entwicklung der Eigenaktivität der Patienten sowie deren Selbstkontrolle unter Einsatz von Persuasion und Gamification.

## Abstract (en)

**Background:**

For the treatment of the symptoms of post-COVID-19 syndrome, no causal therapy is currently widely recommended according to evidence-based criteria. The overarching goal of the intervention study over a 3-year period (Q1-2021–Q4-2023) was to evaluate the changes in the key symptoms of fatigue and sensorimotor instability through individualized stress-controlled training therapy and through intensified cognitive behavioral therapy.

**Material and methods:**

In the 3‑year period 407 vaccinated nucleocapsid positive patients were treated at the Post-COVID-19 Center Lausitz (Senftenberg). In 78 (around 19%) fatigue/immunometabolic depression and sensorimotor instability were identified as the leading syndromes. The evaluation of the individualized stress-controlled training therapy was based on the specific post-COVID-19 syndrome and motor fatigue parameters. The secondary psychosomatic syndrome was assessed using cognitive fatigue parameters and cognitive behavioral therapy instruments. The investigation of -parameters influencing behavior took place in Q2-2023–Q4-2023 with a guide-supported qualitative interview among the participants.

**Results:**

The post-COVID-19 key symptoms “fatigue,” “sensorimotor instability,” “neuropsychiatric symptoms,” “cardiac/autonomic dysfunction,” and “pain” improved significantly in the overall cohort and in the gender-specific analysis. A deterioration occurred in “secondary psychosomatic symptoms”. A therapeutic effect was demonstrated for all motor fatigue parameters for the entire cohort using the Cohen’s d value. An intensification of cognitive behavioral therapy achieved positive effects through an increasing development of the patients’ own activity and their self-control using persuasion and gamification.

Fatigue ist ein sehr häufiges und leitendes Syndrom von Post-COVID-19. In diesem Beitrag werden die Ergebnisse einer neurologisch kontrollierten Konversion durch den Einsatz einer individualisierten belastungsgesteuerten Trainingstherapie sowie einer intensivierten kognitiven Verhaltenstherapie zur Verbesserung der Post-COVID-Leitsymptome, der motorischen und kognitiven Fatigability vorgestellt. Männer erzielten durch einen schnelleren Start der Trainingstherapie positivere Ergebnisse, was Auswirkungen auf die Reduzierung vorhandener Teilhabestörungen sowie die Wiederherstellung der Arbeitsfähigkeit hatte.

Im Rahmen der SARS-CoV-2-Pandemie haben sich große Teile der Bevölkerung mit einem immunogenen Virus angesteckt, der gesundheitliche (Langzeit‑)Folgen unterschiedlicher Schwere und Dauer verursachte. In der Fachliteratur sowie der AWMF-S1-Leitlinie hat sich der Begriff Post-COVID-Syndrom (PCS) durchgesetzt [6, 13]. Anhand der Delphi-Konsensus-Methode hat die WHO (World Health Organization) das PCS wie folgt definiert [18, 23]:

- die Symptome müssen noch nach 12 Wochen (3 Monate) nach einer SARS-CoV-2-Infektion bestehen und mindestens 2 Monate andauern,
- keine andere ätiologische Erklärung kann gegeben werden,
- der Verlauf kann persistierend, rezidivierend oder fluktuierend sein.

Ein sehr häufiges und oft leitendes Post-COVID-19-Syndrom ist Fatigue. Die Fatigue wird durch eine subjektiv oft stark einschränkende, zu den vorausgegangenen Anstrengungen unverhältnismäßige, durch Schlaf nicht aufzuhebende körperliche (insb. motorische), kognitive und/oder psychische Erschöpfung charakterisiert [5, 8, 9, 13, 16, 18]. Im Zentrum stehen die bei geringer objektiver Belastung durch Leistungsanforderungen ausgelösten Beanspruchungsreaktionen somatischer, kognitiver und emotionaler Art, die in ihrem Ausmaß im Vorfeld für den Betroffenen oft nicht absehbar sind (Crash bzw. postexertionelle Malaise [PEM]). Aus Sicht der Psychiatrie, werden die bei immunologisch getriggerten Patienten auftretenden depressiven Erkrankungen als immunometabolische Depression bezeichnet, da sie für eine klassische Depression eine eher atypische Symptomkonstellation, wie z. B. Fatigue, aufweisen [1].

Mit Fatigue (Neurologie, Neurokardiopulmologie) und immunometabolischer Depression (Psychiatrie, Psychosomatik, Immunologie) kommt es zum Brückenschlag der betroffenen Fachgebiete bei der Untersuchung immunologisch getriggerter energiedefizitär bedingter Netzwerkstörungen in verschiedenen Organsystemen. Das Nervensystem als Gesamtheit ist hierbei am häufigsten betroffen [2]. Akzeptiert ist, dass einerseits im Rahmen der Prozessphasen von COVID-19 eine Vielfalt von Organschädigungen, bspw. in Lunge, Herz, Hirn oder peripherem Nervensystem, auftreten können (Abb. 1) sowie andererseits psychische Komorbiditäten in individuell unterschiedlichen Kombinationen für die Entstehung von Fatigue bedeutsam sind [13]. Als pathophysiologische Mechanismen werden diskutiert [10]:

- Viruspersistenz (nicht wahrscheinlich),
- anhaltende Überaktivierung des Immunsystems, einschl. Autoimmunphänomene,
- fortschreitende Thrombusbildung im Mikrogefäßsystem und anderweitige Gewebehypoxämie.

**Abb. 1:**
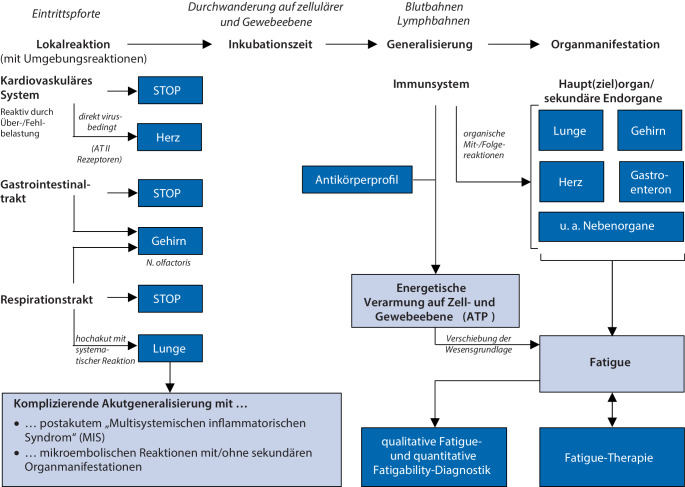
Prozessphasen von COVID-19/Post-COVID-19

Für die Behandlung der Symptome des Post-COVID-19-Syndroms wird derzeit keine kausale Therapie nach evidenzbasierten Kriterien breit empfohlen. Die S2k-Leitlinie der Deutschen Gesellschaft für Neurologie (DGN) empfiehlt, dass Post-COVID-19-Betroffene mit sensorischen, sensomotorischen, kognitiven und/oder emotionalen Veränderungen einer adäquaten neurologischen Evaluation und bei Bedarf einer neurorehabilitativen Versorgung zugeführt werden sollen. Größte Effekte auf Fatigue werden bei Gleichgewichtstraining, gefolgt von auf Fatigue ausgelegter kognitiver Verhaltenstherapie sowie motorischen Übungstherapien gesehen [4].

Eine multimodale Therapie für die Post-COVID-19-Symptome Fatigue, Belastungsintoleranz, sensomotorische Instabilität und organbezogene Syndromatik sollte eine Symptomlinderung, die Vermeidung der Chronifizierung, eine Reduzierung vorhandener Teilhabestörungen sowie eine Wiederherstellung der Arbeitsfähigkeit umsetzen [14, 19]. Empfohlen wird eine an die individuelle Belastbarkeit angepasste, kontrollierte Durchführung körperlicher und kognitiver Aktivitäten unter Vermeidung einer Überbeanspruchung mit nachfolgender Symptomverschlechterung [13, 18, 20]. Folgende Zielstellungen verband daher die durchgeführte Interventionsstudie:

- Verbesserung der körperlichen Gesundheit,
- Förderung der psychosozialen Gesundheit,
- Aufbau von Teilhabe am Arbeitsleben und der Gesellschaft,
- Erklärung unterschiedlicher Determinanten des Gesundheitsverhaltens der Studienteilnehmer.

Die Evaluation der individualisierten beanspruchungsgesteuerten Trainingstherapie erfolgte anhand der Post-COVID-19-Leitsymptome (Fatigue, sensomotorische Instabilität, kardiopulmonale/autonome Dysfunktion, neuropsychiatrische Symptome, sekundär psychosomatische Symptome und Schmerzen) sowie motorischer Fatigability-Parameter (Berg-Balance Scale, Dynamic Gate Index, Functional Reach Test, Monopedalstand sowie 10-m-Gehtest). Die motorische Fatigability stellt einen objektiv messbaren Indikator für die motorische Performance dar und lässt eine höhere medizinische Bewertung der Therapiewirksamkeit zu [3, 7, 11, 12, 21, 22]. Mit einem leitfadengestützten Interview mit den Interventionsteilnehmern wurden die Wirkungen unterschiedlicher Determinanten auf das Gesundheitsverhalten erklärt.

## Studiendesign und Methodik

### Aufbau

**Abb. 2:**
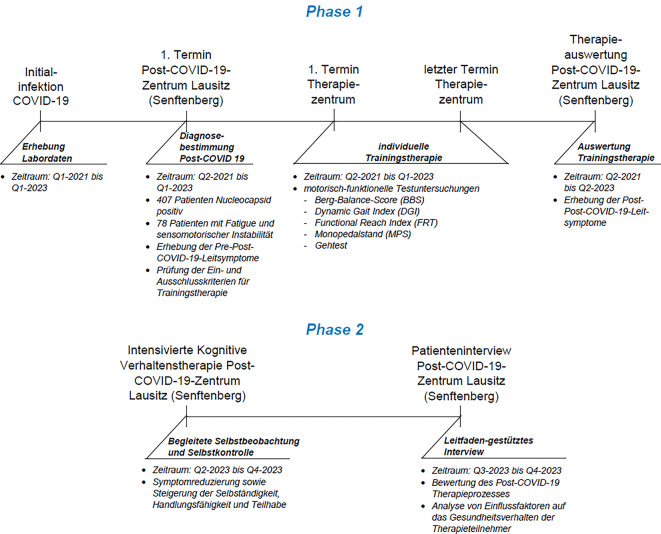
Methodik und Ablauf der Interventionsstudie

Im Post-COVID-19-Zentrum (PCZ) Lausitz (Senftenberg) wurde eine individualisierte, durch Beanspruchungsmessung eingeregelte Trainingstherapie zur Reduzierung von Fatigue und sensomotorischer Instabilität sowie eines sich oft im zeitlichen Post-COVID-19-Verlauf mit Fatigue ergebenden sekundären psychosomatischen Behandlungsbedarfs in Form einer Interventionsstudie umgesetzt (Abb. 2). Der Betrachtungszeitraum beläuft sich von Q1-2021 bis Q4-2023 in zwei Behandlungsphasen:

- *Behandlungsphase 1:* Individualisierte beanspruchungsgesteuerte Trainingstherapie zur Verbesserung der quantifizierten sensomotorischen Parameter und der Post-COVID-19-Leitsymptome (Q2-2021 bis Q2-2023).
- *Behandlungsphase 2: *Intensivierte kognitive Verhaltenstherapie zur Verbesserung von Fatigue und sekundären psychosomatischen Symptomen (Q2-2023 bis Q4-2023) sowie die Erfassung der subjektiven Wirksamkeit der Trainingstherapie mit einem standardisierten Leitfadeninterview in Q3-2023 bis Q4-2023.

### Stichprobe

**Abb. 3:**
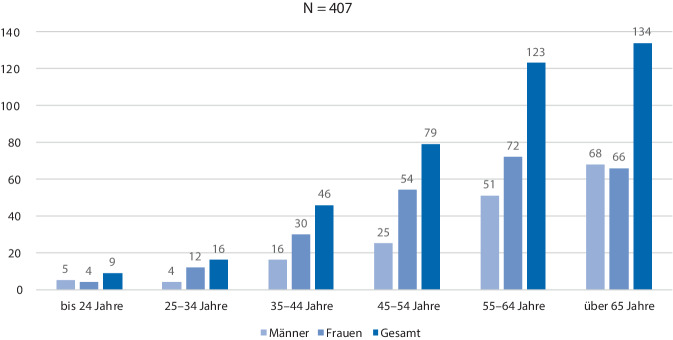
Struktur der nukleokapsidpositiven Patienten

**Abb. 4:**
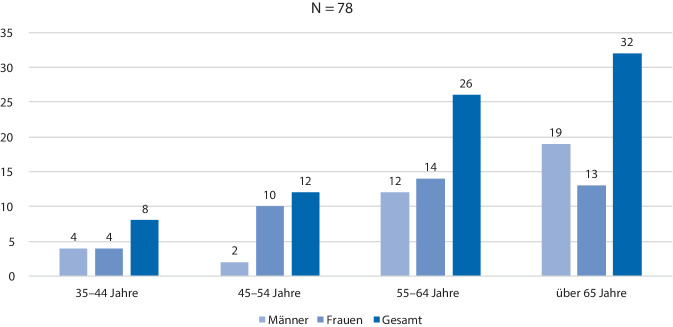
Struktur der Patienten mit Fatigue und sensomotorischer Instabilität

Den Ausgangspunkt für die Bestimmung der Stichprobe der Teilnehmer der Interventionsstudie bildeten 407 geimpfte nukleokapsidpositive Patienten (Abb. 3), die im PCZ Senftenberg vorgestellt wurden. Die Ermittlung des Nukleokapsidproteins erfolgte im PCZ durch eine Blutentnahme und Laboruntersuchung oder im Vorfeld durch einen überweisenden Hausarzt/Facharzt/Krankenhaus im Zeitraum von Q1-2021 bis Q1-2023. In die Interventionsstudie (sensomotorische Trainingstherapie) einbezogen wurden aus der Grundgesamtheit von 407 Patienten unter Beachtung von Ein- und Ausschlusskriterien (Tab. 1) von Q2-2021 bis Q2-2023 im PCZ 78 (rd. 19 %) Post-COVID-19-Patienten mit dem Syndrom „Fatigue“ und „sensomotorische Instabilität“. Sie wurden leitliniengerecht standardisiert diagnostiziert und einer basalen symptomatischen medikamentösen Therapie unterzogen. Basierend auf der WHO-Klassifikation des Verlaufes einer SARS-CoV-2-Infektion konnten 88 % der Interventionsteilnehmer der viralen Phase-ambulant (WHO-CPS (WHO Clinical Progression Score): 1–3) und je 5,5 % der pulmonalen Phase-stationär (WHO-CPS: 4–5) sowie der hyperinflamatorischen Phase-ITS (WHO-CPS: 6–9) zugeordnet werden [8]. Die Patientenstruktur und die Vorerkrankungen sind in Abb. 4 sowie Tab. 2 dargestellt.

**Tab. 1:** Ein- und Ausschlusskriterien der Interventionsstudie

Einschlusskriterien	Ausschlusskriterien
Volljährigkeit	Unüberbrückbare Kommunikationsprobleme
Vollständige Einwilligungs- und Geschäftsfähigkeit	Vestibuläre Dysfunktion (Video-Kopfimpulstest)
Studienaufklärung	Herzschrittmacher
Auf Einverständnis bestehende Abgabe einer schriftlichen Einverständniserklärung	Nichtkompensationsfähige visuelle Einschränkungen
	Orthopädische Defizite der unteren Extremitäten
	Unfähigkeit, selbstständig ohne Hilfsmitteleinsatz zu stehen

**Tab. 2:** Vorerkrankungen der Patienten mit sensomotorischer Instabilität

Organische (somatische) Syndromatik	Psychische Syndromatik
Hypertonie/Herzkreislauf: 12 Personen	Depression: 4 Personen
Chronische Schmerzstörung: 10 Personen	Angststörungen: 2 Personen
Metabolisches Syndrom: 8 Personen	
Epilepsie/Lähmungserscheinungen: 4 Personen	

Alle Patienten waren volljährig und gaben eine schriftliche Einverständniserklärung vor Aufnahme in die Studie ab. Das umgesetzte Studiendesign sowie das eingesetzte computergesteuerte Trainingssystem wurden von der Ethikkommission der TU Dresden genehmigt (Referenznummern EK 378092016, EK 356092017).

### Behandlungsphase 1: Individualisierte beanspruchungsgesteuerte Trainingstherapie

Die Trainingstherapie in Form einer Blended Therapy erfolgte in einem zum PCZ zugehörigen Trainingszentrum sowie in der Häuslichkeit, um eine ausreichende Trainingsdichte (mind. 2 Trainingseinheiten pro Woche) zu erreichen. Das Training in der Häuslichkeit wurde durch die Patienten dokumentiert und im Trainingszentrum ausgewertet. Zum Einsatz kam ein computergestütztes Trainingssystem. Patienten, bei denen in der Häuslichkeit kein computergesteuertes Trainingssystem eingesetzt wurde, führten die Übungen eigenständig mit Messung der Pulsfrequenz über eine Smartwatch sowie Dokumentation der Ergebnisse durch.

Für das vom PCZ eingesetzte computergesteuertes Trainingssystem war im Vorfeld im Zusammenwirken mit der Medizinischer Fakultät der TU Dresden und dem Institut für Medizinische Informatik der Brandenburgischen Technischen Universität Cottbus/Senftenberg (BTU) seit 2018 eine Trainingstherapie entwickelt worden, deren Belastungssteuerung auf eine in Echtzeit mögliche Beanspruchungsmessung des betroffenen Patienten begründet ist [15, 17]. Hierdurch wurde es möglich, bei immunologisch und/oder anderweitig beanspruchten energetisch verarmten Patienten mit Fatigue/immunometabolischer Depression [1] dosierte Trainingsreize zu setzen, die bis dahin als kontraindiziert galten. Ohne diesen Steuerungsmechanismus kommt es zu Crash/PEM und somit zu einer weiteren starken Verschlechterung der Leistungsfähigkeit des Patienten (z. B. bei Multipler Sklerose, in der Hämatoonkologie und aktuell im Rahmen der Immuntherapie von metastasierenden Tumoren).

Die Echtzeitkontrolle von Beanspruchungsparametern wurde so für das Einregeln der individuellen Trainingsbelastung eine conditio sine qua non, die auch bei hohen Behandlungszahlen z. B. unter Pandemiebedingungen realisierbar ist. Das Ausmaß und die Qualität einer Handlungskontrolle wurden in vorauslaufenden Arbeiten als ein in Echtzeit zu bestimmender kognitiver Parameter untersucht, eingeführt und als Routineparameter qualitativ überprüft.

Eine erfolgreiche Handlungskontrolle setzt eine effektive und effiziente Interaktion aller posturalen Systeme voraus, die sowohl aktiv als auch reaktiv mittels muskulärer Kräfte den Körper im Gleichgewicht halten. Sie basiert auf der Verarbeitung von sensorischen Wahrnehmungen (vestibulär, visuell, propriozeptiv etc.), die sowohl auf spinaler als auch supraspinaler Ebene (Kortex, insb. Subkortex und Zerebellum) lokalisiert sind und der mentalen Vorwegnahme zukünftiger Bewegungsabläufe (Antizipation). Auf Grundlage der Afferenzen wird im anschließenden motorischen Prozess unter Einbeziehung antizipatorischer und adaptiver Vorgänge eine posturale Reaktion in Form einer gezielten Ansteuerung der stabilisierenden Muskulatur generiert.

**Abb. 5:**
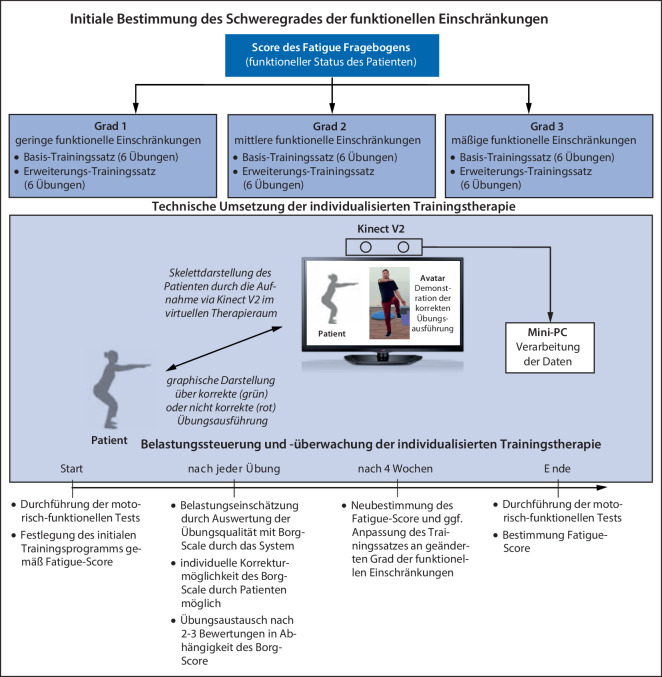
Systemaufbau und Methodik der individualisierten belastungsgesteuerten Trainingstherapie

Der Systemaufbau sowie die eingesetzte Methodik der individualisierten belastungsgesteuerten Trainingstherapie basiert auf drei Prozessschritten (Abb. 5):

1. initiale Bestimmung des Schweregrades der funktionellen Einschränkungen,
2. technische Umsetzung der individualisierten Trainingstherapie,
3. Belastungssteuerung durch Überwachung der individuellen Beanspruchung während der Trainingstherapie.

### Behandlungsphase 2: Intensivierte kognitive Verhaltenstherapie

In Phase 2 wurden die Therapieteilnehmer mit „sekundären psychosomatischen Symptomen“ aus Phase 1 übernommen. Im Erleben des Post-COVID-19-Betroffenen kann sich im Zeitverlauf (auch im Therapieverlauf am PCZ) eine zunehmende Gedankendysfunktionalität aus einer im beruflichen und im häuslichen Alltag erlebten Belastungsinsuffizienz entwickeln [2, 14]. Mit der Intensivierung der kognitiven Verhaltenstherapie i. S. einer Erhöhung der Eigenaktivität der Patienten (auch in der Häuslichkeit), wurden im Zeitraum Q2-2023 bis Q4-2023 Veränderungen der sekundären psychosomatischen Syndromatik untersucht (kognitive Fatigability-Parameter). Des Weiteren fand im Zeitraum Q3-2023 bis Q4-2023 ein leitfadengestütztes qualitatives Interview mit allen Therapieteilnehmern statt.

## Ergebnisse

Die Ergebnisse der Gesamtbetreuung in der Phase 1 des Post-COVID-19-Therapieprozesses sind in Tab. 3 dargestellt. Es gab keine starken geschlechtsspezifischen Schwankungen. Abweichungen bestehen in einzelnen Teilphasen und haben Auswirkungen auf das Therapieoutcome.

**Tab. 3:** Zeitverlauf Post-COVID-19-Therapieprozess (Phase 1)^a^

	Zeitraum Inititalinfektion‑1. Termin Post-COVID-19-Zentrum	Zeitraum 1. Termin Post-COVID-19-Zentrum‑1. Termin Therapiezentrum	Zeitraum 1. Termin Therapiezentrum letzter Termin Therapiezentrum	Zeitraum letzter Termin Therapiezentrum-Therapieauswertung im PCZ
Mittelwert	Gesamt: 85 Tage	Gesamt: 15 Tage	Gesamt: 99 Tage	Gesamt: 33 Tage
	Männer: 77 Tage	Männer. 8 Tage	Männer: 106 Tage	Männer: 36 Tage
	Frauen: 93 Tage	Frauen: 22 Tage	Frauen: 93 Tage	Frauen: 29 Tage
Median	Gesamt: 76 Tage	Gesamt: 6 Tage	Gesamt: 84 Tage	Gesamt: 27 Tage
	Männer: 67 Tage	Männer: 7 Tage	Männer: 84 Tage	Männer: 30 Tage
	Frauen: 88 Tage	Frauen: 7 Tage	Frauen: 75 Tage	Frauen: 19 Tage
Standardabweichung	Gesamt: 51 Tage	Gesamt: 37 Tage	Gesamt: 59 Tage	Gesamt: 29 Tage
	Männer: 45 Tage	Männer: 10 Tage	Männer: 64 Tage	Männer: 28 Tage
	Frauen: 56 Tage	Frauen: 49 Tage	Frauen: 52 Tage	Frauen: 29 Tage

^a^Gesamtkohorte *N* = 78, Männer *N* = 37, Frauen *N* = 41

*PCZ* Post-COVID-Zentrum

### Post-COVID-19-Leitsymptome

**Abb. 6:**
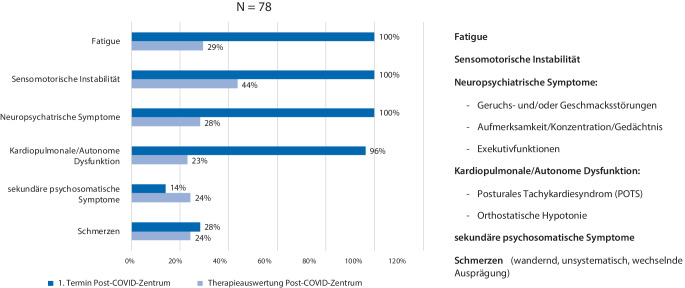
Entwicklung der Post-COVID-Leitsymptome (Phase 1)

Die Gesamtbetrachtung mit *N* = 78 (Abb. 6 und Tab. 4) ergab folgende Ergebnisse:

- Verbesserungen von „Fatigue“ um rd. 71 %, „sensomotorische Instabilität“ um rd. 56 %, „neuropsychiatrische Symptome“ um rd. 72 %, „kardiopulmonale/autonome Dysfunktion“ um rd. 76 % sowie „Schmerzen“ um rd. 14 %,
- Verschlechterung der „sekundären psychosomatischen Symptomen“ um rd. 72 %.

**Tab. 4:** Entwicklung der Post-COVID-19-Leitsymptome (Phase 1)

Gesamtkohorte (*N* = 78)	–	Fatigue	Sensomotorische Instabilität	Kardiopulmonale/autonome Dysfunktion	Neuropsychiatrische Symptome	Sekundär psychosomatische Symptome	Schmerzen
	1. Termin Post-COVID-19-Zentrum	*N* = 78 (100 %)	*N* = 78 (100 %)	*N* = 76 (97 %)	*N* = 78 (100 %)	*N* = 11 (14 %)	*N* = 22 (28 %)
	Therapieauswertung	*N* = 23 (29 %)	*N* = 34 (47 %)	*N* = 18 (23 %)	*N* = 22 (28 %)	*N* = 19 (24 %)	*N* = 19 (24 %)
	*Verbesserung*	*N* *=* *55 (71* *%)*	*N* *=* *44 (53* *%)*	*N* *=* *58 (76* *%)*	*N* *=* *56 (72* *%)*	*N* *=* *−8 (−72* *%)*	*N* *=* *3 (14* *%)*
Männer (*N* = 37)	1. Termin Post-COVID-19-Zentrum	*N* = 37 (100 %)	*N* = 37 (100 %)	*N* = 36 (97 %)	*N* = 37 (100 %)	*N* = 6 (16 %)	*N* = 10 (27 %)
	Therapieauswertung	*N* = 6 (16 %)	*N* = 15 (41 %)	*N* = 6 (16 %)	*N* = 10 (27 %)	*N* = 4 (11 %)	*N* = 10 (27 %)
	*Verbesserung*	*N* *=* *31 (84* *%)*	*N* *=* *22 (59* *%)*	*N* *=* *30 (83* *%)*	*N* *=* *27 (73* *%)*	*N* *=* *2 (33* *%)*	*N* *=* *0 (0* *%)*
Frauen (*N* = 41)	1. Termin Post-COVID-19-Zentrum	*N* = 41 (100 %)	*N* = 41 (100 %)	*N* = 40 (98 %)	*N* = 41 (100 %)	*N* = 5 (12 %)	*N* = 12 (29 %)
	Therapieauswertung	*N* = 17 (41 %)	*N* = 19 (46 %)	*N* = 12 (29 %)	*N* = 12 (29 %)	*N* = 15 (37 %)	*N* = 9 (22 %)
	*Verbesserung*	*N* *=* *24 (59* *%)*	*N* *=* *22 (54* *%)*	*N* *=* *28 (70* *%)*	*N* *=* *29 (71* *%)*	*N* *=* *−10 (−200* *%)*	*N* *=* *3 (25* *%)*

Bei den Männern (*N* = 37) verbesserten sich bis auf die „Schmerzen“ (keine Verbesserung) alle Werte gegenüber den Frauen. Ein Grund kann in den schnelleren Prozessabläufen (Tab. 3) im Zeitraum „Initialinfektion‑1. Termin PCZ“ sowie „1. Termin PCZ‑1. Termin Therapiezentrum“ gesehen werden. Eine sehr starke Verschlechterung erzielten Frauen bei den „sekundär psychosomatischen Symptomen“. Als ursächlich kann der langsamere Prozessablauf der o. g. Zeiträume gegenüber den Männern (Tab. 3), die nicht rechtzeitige Einschätzung der (Rest‑)Leistungsfähigkeit sowie ausweichendes Verhalten, vorzugsweise bei hyperdynamen jungen Frauen in Form dysfunktionaler psychischer Reaktionen, angesehen werden.

### Motorische Fatigability-Parameter

Für die Bewertung der Therapiewirksamkeit wurde der Wilcoxon-Test durchgeführt, da keine Normalverteilung in den Prä- und Postdatensätzen vorlag. Der konkrete Wirkungseffekt der Trainingstherapie auf den jeweiligen Fatigibility-Parameter erfolgt anhand des Cohens d-Wertes.

In der Gesamtbetrachtung (*N* = 78) wurde für alle motorischen Fatigability-Parameter ein therapeutischer Effekt nachgewiesen (Tab. 5). Eine mittlere Effektstärke erzielte der Functional Reach Test (FRT). Für alle anderen motorischen Fatigability-Parameter ergaben sich geringe Effektstärken.

**Tab. 5:** Entwicklung der motorischen Fatigability-Parameter (Phase 1) – gesamt und geschlechtsspezifisch

Gesamtkohorte (*N* = 78)	–	Berg-Balance-Score (BBS)	Dynamic Gait Index (DGI)	Functional Reach Test (FRT)	Monopedalstand (MPS)	10-m-Gehtest
	Prä	MW = 48,5	MW = 21,8	MW = 32 cm	MW = 88,8 sec	MW = 6,6 sec
	Post	MW = 51	MW = 22,9	MW = 36,7 cm	MW = 100,8 sec	MW = 6,3 sec
	*Signifikanz* (Wilcoxon-Test 2‑seitig)	*<* *0,001*	*<* *0,001*	*<* *0,001*	*0,036*	*0,017*
	*Verbesserung*	*5,1* *%*	*4,6* *%*	*14,7* *%*	*13,6* *%*	*4,5* *%*
	*Effektstärke* (Cohens d)	*−0,48 *(gering)	*−0,42 *(gering)	*−0,61 *(mittel)	*−0,22 *(gering)	*0,201 *(gering)
Männer (*N* = 37)	Prä	MW = 49	MW = 21,9	MW = 33,7 cm	MW = 75 sec	MW = 6,21 sec
	Post	MW = 51,7	MW = 23	MW = 37,7 cm	MW = 96,8 sec	MW = 6,24 sec
	*Signifikanz* (Wilcoxon-Test 2‑seitig)	*<* *0,001*	*0,02*	*0,001*	*0,044*	*0,551*
	*Verbesserung*	*5,5* *%*	*5* *%*	*11,8* *%*	*29* *%*	*0,5* *%*
	*Effektstärke* (Cohens d)	*−0,7 *(mittel)	*−0,38 *(gering)	*−0,56 *(mittel)	*−0,30 *(gering)	*0,02 *(sehr gering)
Frauen (*N* = 41)	Prä	MW = 48,1	MW = 21,8	MW = 30,4 cm	MW = 99,3 sec	MW = 7,0 sec
	Post	MW = 50,5	MW = 22,8	MW = 35,8 cm	MW = 104,4 sec	MW = 6,3 sec
	*Signifikanz* (Wilcoxon-Test 2‑seitig)	*0,017*	*0,07*	*<* *0,001*	*0,382*	*0,05*
	*Verbesserung*	*5* *%*	*4,6* *%*	*15,1* *%*	*5,1* *%*	*10* *%*
	*Effektstärke* (Cohens d)	*−0,38 *(gering)	*−0,45 *(gering)	*−0,64 *(mittel)	*−0,12 *(sehr gering)	*0,4 *(gering)

*MW* Mittelwert

Bei den Männern konnte für den 10-m-Gehtest kein Therapieeffekt nachgewiesen werden. Mittlere Effektstärken erzielten der Berg Balance Score (BBS) sowie der FRT. Geringe Effektstärken ergaben sich für den Dynamic Gait Index (DGI) und den Monopedalstand (MPS).

Im Rahmen der Frauenkohorte (*N* = 41) ergaben sich für den Dynamic Gait Index (DGI) und den Monopedalstand (MPS) keine Therapieeffekte. Eine mittlere Effektstärke erzielte der FRT. Geringe Effektstärken ergaben sich für den BBS sowie 10-m-Gehtest.

### Kognitive Fatigability-Parameter

In Tab. 6 ist die Entwicklung der Studienteilnehmer mit Fatigue sowie sekundär psychosomatischen Symptomen nach dem Ende der Phase 1 und der sich anschließenden intensivierten kognitiven Verhaltenstherapie über 3 Quartale (Phase 2: Q2-2023 bis Q4-2023) dargestellt. Sowohl in der Gesamtbetrachtung (Verbesserung rd. 53 %) als auch in der geschlechtsspezifischen Analyse (Verbesserung Männer: 50 %; Verbesserung Frauen: rd. 53 %) ist die positive Wirkung der Selbstbeobachtung mit der begleiteten Selbstkontrolle ersichtlich.

**Tab. 6:** Entwicklung der sekundären psychosomatischen Syndromatik in Phase 2 unter Intensivierung der kognitiven Verhaltenstherapie – gesamt und geschlechtsspezifisch

Sekundär psychosomatische Syndromatik			
	Gesamt	Männer	Frauen
Therapieauswertung am PCZ (Ende Phase 1) = Beginn der Intensivierung kognitive Verhaltenstherapie (Start Phase 2)	*N* = 19 (100 %)	*N* = 4	*N* = 15
Nach 3 Quartalen intensivierter kognitiver Verhaltenstherapie (Ende Phase 2)	*N* = 9 (47,4 %)	*N* = 2	*N* = 7
*Verbesserung*	*N* *=* *10 *(52,6 %)	*N* *=* *2 *(50 %)	*N* *=* *8 (53,3* *%)*

### Leitfadengestütztes Interview

Die Erfassung der Wirkungen unterschiedlicher Determinanten auf das Gesundheitsverhalten der Studienteilnehmer erfolgte in Q3-2023 und Q4-2023 mit einem leitfadengestützten Interview. Mit den Ergebnissen wurden folgende drei Fragen für Empfehlungen zum Management einer neurologisch kontrollierten Konversion beantwortet:

F 1: Haben die persönliche Bindung zu einem Arzt und/oder Therapeuten sowie die regelmäßige Anpassung und Kontrolle von Therapiezielen einen Einfluss auf das Gesundheitsverhalten?

- Persönliche Arzt-Therapeutenbindung bei der Trainingstherapie
  - „zum Arzt“: 31 Nennungen
  - „zum Physiotherapeuten“: 25 Nennungen
  - „zum Trainingstherapeuten“: 21 Nennungen
  - „zu anderen Therapeuten“: 8 Nennungen
  - „keine Aussage“: 7 Teilnehmer
- Einfluss individuell angepasster und kontrollierter Therapieziele auf die Therapietreue
  - „ja“: 28 Nennungen
  - „nein“: 8 Nennungen
  - „keine Aussage“: 8 Teilnehmer

F 2: Welche Ängste haben Patienten mit der Post-COVID-19-Erkrankung?

- „Angst vor sich verschlechternden körperlichen Auswirkungen“: 26 Nennungen
- „Angst, dem Alltag nicht gewachsen zu sein“: 25 Nennungen
- „Angst vor geistigen Verschlechterungen“: 11 Nennungen
- „Angst vor Schmerzen“: 9 Nennungen
- „keine Aussage“: 5 Teilnehmer

F 3: Welche Faktoren haben am meisten im Genesungsprozess geholfen?

- Training und Bewegung: Koordinationstraining, Physiotherapie, Reha, Sport und Trainingstherapie
- Entspannung und soziale Kontakte: Ruhe und kein Stress sowie Familie und Freunde
- Medizinische Unterstützung: Zusammenarbeit mit PCZ sowie Medikamente gegen Konzentrationsschwäche

## Diskussion/Limitationen/Ausblick

Im Rahmen der neurologisch kontrollierten Konversion von Post-COVID-19-Patienten mit Fatigue und sensomotorischer Instabilität konnte nachgewiesen werden, dass durch eine den medizinischen Hauptprozess am Post-COVID-19-Zentrum begleitende individualisierte beanspruchungsgesteuerte Trainings- und in Ansätzen auch kognitive Verhaltenstherapie (Phase 1) sowie mit einer intensivierten kognitiven Verhaltenstherapie mit Selbstkontrollschwerpunkt in der Häuslichkeit (Phase 2) die Post-COVID-19-Leitsymptome „Fatigue“, „sensomotorische Instabilität“, „neuropsychiatrische Symptome“, „kardiopulmonale/autonome Dysfunktion“, „sekundäre psychosomatische Symptome“ sowie „Schmerzen“ reduziert werden konnten.

Mit der umgesetzten individualisierten beanspruchungsgesteuerten Trainingstherapie in Form einer Blended Therapy, d. h. der Kombination aus einem computerbasierten Trainingssystem und einem Vor-Ort-Setting, konnte anhand der motorischen Fatigability-Parameter nachgewiesen werden, dass sich Fatigue und sensomotorische Instabilität gut behandeln lassen. Das eingesetzte computerbasierte Trainingssystem beinhaltete dabei folgende Funktionalitäten:

- Beobachtung und Echtzeitauswertung des Trainings auf dem Bildschirm,
- belastungsgesteuerte Beanspruchung,
- Steigerung von Behandlungsadhärenz, Motivation und Therapieerfolg durch Gamification-Elemente,
- Therapieplanung in Abhängigkeit des Trainingsstandes sowie Förderung von Eigenaktivität und Selbstbestimmung.

Für das Realisieren eines individualisierten Behandlungserfolgs koordinierte das PCZ als Orchestrator einen strukturierten Informationsaustausch und die Prozesse zwischen den einzelnen Akteuren. Gleichzeitig lernte der Patient am PCZ Behandlungselemente kennen, die seine Eigenaktivität stärken, die er auf dem weiteren Pathway nutzen kann und die ihn anhaltend verhaltensmodifizierend beeinflussen.

Durch die im Zusammenwirken der Therapie am PCZ und in der Häuslichkeit realisierte hybride Trainingstherapie (Blended Therapy) ist es möglich geworden, eine in den Vorjahren entwickelte und praxiserprobte internet- und mobilebasierte Intervention (IMI) zu nutzen. Es wurden inhaltliche und strukturelle Verknüpfungen anderer medizinischer Fachgebiete durch Einbindung von Medizininformatik und künstlicher Intelligenz realisiert. Um nachhaltige, gesundheitsfördernde Lebensstilveränderungen, Verbesserungen der Teilhabe sowie Lebensqualität zu erreichen, bedurfte es einer modernen Umsetzung der kognitiven Verhaltenstherapie über klassische Therapie- und Rehabilitationsphasen hinaus.

Im Rahmen der vorliegenden Studienergebnisse standen – bezogen auf Post-COVID-19 – nur monozentrisch erhobene Daten zur Verfügung, da zum Ausbruch der Pandemie nur auf die bereits bestehenden praxisrelevanten Verknüpfungen zurückgegriffen werden konnte (personelle und technische Voraussetzungen). Aktuell ist eine routinemäßige Umsetzung an weiteren miteinander verbundenen Therapiezentren erfolgt (Hoyerswerda, Cottbus, Dresden u. a.). Die Nachhaltigkeit derartiger Strukturen ist bspw. durch eine Verknüpfung mit der modernen Immuntherapie in der Onkologie bereits realisiert.

## Fazit für die Praxis

- Ein effizientes Patientenmanagement, die ganzheitliche Integration und Koordination der einzelnen Akteure im Behandlungsprozess, ein systemorientiertes multimodales Behandlungskonzept (vom Krankenhaus bis in die Häuslichkeit), ein konsequentes Selbstmanagement mit Entspannungsübungen und Pacing sowie die Umsetzung einer hohen Therapiedichte führt zu Verbesserungen von Post-COVID-19-Leitsymptomen, der motorischen und kognitiven Fatigability-Parameter sowie der Vermeidung von Prozessstörungen in der Therapie.
- Für einen erfolgreichen Outcome ist die Definition von Schwerpunktpathologie sowie deren Gliederung nach Alter und Geschlecht mit Schlussfolgerungen für die Therapie und den hierfür notwendigen Personaleinsatz vorzunehmen.
- Der Einsatz einer modernen Belastungs- und Beanspruchungsdiagnostik mit einer Belastungssteuerung, erlaubt die Vermeidung eines Crash-Phänomens nach inadäquater Belastungssteuerung.
- Die Intensivierung der Verhaltenstherapie bei Kohorten mit besonderer Anfälligkeit für Sekundärpathologie kann mit begleiteter Selbstbeobachtung und Selbstkontrolle langfristig positive gesundheitsfördernde Verhaltenskonsequenzen beim Patienten erzielen.
- In der Routinepraxis muss die Gültigkeit der eingesetzten Testverfahren, insb. bei einer Zusammenhangsbegutachtung, erfolgen.

## Data Availability

Die verwendeten Studiendaten liegen in elektronischer Form vor.

## References

[1] Aichholzer M et al (2021) Immunometabolische Depression – ein neues Target für die Präzisionsmedizin in der Psychiatrie? Neuro Aktuell 2021:22–28

[2] Beck JS (2013) Praxis der Kognitiven Verhaltenstherapie. Beltz, Weinheim

[3] Behrens M, Broscheid KC, Schega L (2021) Taxonomie und Determinanten Motorischer Performance Fatigability bei Multipler Sklerose. NR 27(1):3–12

[4] Berlit P et al (2022) Neurologische Manifestationen bei COVID-19. Leitlinien für Diagnostik und Therapie in der Neurologie. AMWF-Registernummer: 030/144

[5] Buchberger B et al (2022) Post-Corona-Fatigue – das bekannte Bild in neuem Gewand? Onkologe 28:340–346 10.1007/s00761-022-01102-1 PMC8853121 35194336

[6] Bundesärztekammer (2022) Post-COVID-Syndrom (PCS). Dtsch Ärztebl

[7] Dettmers Ch (2023) Motorische Rehabilitation von Fatigue. AMWF-Registernummer 030/123, S 87–91

[8] Feldt T et al (2023) Hinweise zur Erkennung, Diagnostik und Therapie von Patienten mit COVID-19. STAKOB Geschäftsstelle am Robert-Koch-Institut, Berlin

[9] Flachenecker P (2021) Definition, Epidemiologie und ätiologische Faktoren. Hippocampus, Bad Honnef, S 7–23

[10] Fleischer et al (2022) Post-COVID-19 Syndrome is Rarely Associated with Damage of the Nervous System: Findings from a Prospective Observational Cohort Study in 171 Patients. Neurol Ther 11:1637–1657 36028604 10.1007/s40120-022-00395-z PMC9417089

[11] Kluger BM et al (2013) Fatigue and fatigability in neurologic illness: proposal for a unified taxonomy. Neurology 80(4):409–416 23339207 10.1212/WNL.0b013e31827f07be PMC3589241

[12] Kluger BM et al (2013) Fatigue and fatigability in neurologicillnesses: proposal for aunified taxonomy. Neurology 80:409–416 23339207 10.1212/WNL.0b013e31827f07be PMC3589241

[13] Koczulla A et al (2022) AWMF S1-Leitlinie Long/Post-COVID. AMWF-Registernummer: 020-027

[14] Kupferschmitt A et al (2022) Nicht nur multimodal, sondern auch interdisziplinär: Ein Konzept für fächerübegreifende Zusammenarbeit in der Rehabilitation des Post-COVID-Syndroms. Psychother Psychosom Med Psychol 73:34–41 35605967 10.1055/a-1838-3055

[15] Lohse P et al (2020) Optimierung der Langzeitbetreuung von neurologischen Patienten durch internet- und mobile-basierte Interventionen. Fortschr Neurol Psychiatr 88(8):1028–1073 10.1055/a-1028-7073 32303076

[16] Lorenzen H (2010) Fatigue Management. Umgang mit chronischer Müdigkeit und Erschöpfung. Schulz-Kirchner, Idstein

[17] Reichmann H et al (2018) Dynamische Posturographie zur Quantifizierung der posturalen Kontrolle. Akt Neurol 45(10):625–635

[18] Renz-Polster H, Scheibenbogen C (2022) Wenn COVID nicht aufhört: Post-COVID-Verläufe mit Fatigue und Belastungsintoleranz. Dtsch Medizinische Wochenschrift 147:1320–1329 10.1055/a-1849-8953 36195090

[19] Renz-Polster H, Scheibenbogen C (2022) Post-COVID-Syndrom mit Fatigue und Belastungsintoleranz: Myalgische Enzephalomyelitis bzw. Chronisches Fatigue-Syndrom. Inn Med 63:830–839 10.1007/s00108-022-01369-x PMC9281337 35925074

[20] Rutsch M, Deck R (2023) Medizinische Rehabilitation bei Post-COVID-Syndrom – gesundheitliche und berufliche Veränderungen im Zeitverlauf. Dtsch Rentenversicher 2023:27–51

[21] Seamon BA, Harris-Love MO (2016) Clinical Assessment of Fatigability in Multiple-Sclerosis: A Shift from Perception to Performance. Front Neurol 7:194 27872608 10.3389/fneur.2016.00194 PMC5098192

[22] Tegenthoff M et al (2022) Neurologisch-psychiatrische Begutachtung des Post-COVID-Syndroms. Nervenarzt 8:804–811 10.1007/s00115-022-01292-4 PMC9017073 35438301

[23] World Health Organization (2021) A clinical case definition of post COVID-19 condition by a Delphi consensus. www.who.int/publications/i/item/WHO-2019-nCoV-Post_COVID-19_condition-Clinical_case_definition-2021.1. Zugegriffen: 22. Juni 2022 10.1016/S1473-3099(21)00703-9 PMC8691845 34951953

